# Identification of parathyroid hormone-related protein-derived peptides immunogenic in human histocompatibility leukocyte antigen-A24^+^ prostate cancer patients

**DOI:** 10.1038/sj.bjc.6601960

**Published:** 2004-06-15

**Authors:** A Yao, M Harada, S Matsueda, Y Ishihara, H Shomura, M Noguchi, K Matsuoka, I Hara, S Kamidono, K Itoh

**Affiliations:** 1 Department of Immunology, Kurume University School of Medicine, Fukuoka, Japan; 2 Department of Urology, Kurume University School of Medicine, Fukuoka, Japan; 3 Division of Urology, Department of Organs Therapeutics, Faculty of Medicine, Kobe University Graduate School of Medicine, Hyogo, Japan

**Keywords:** prostate cancer, parathyroid hormone-related protein (PTHrP), cytotoxic T lymphocyte, peptide, HLA-A24

## Abstract

Parathyroid hormone-related protein (PTHrP) is a key factor in the development of bone metastases, which are a major barrier in treating prostate cancer patients. In this study, we attempted to identify PTHrP-derived peptides immunogenic in human histocompatibility leukocyte antigen (HLA)-A24^+^ prostate cancer patients. Among four different PTHrP peptides carrying the HLA-A24 binding motif, both the PTHrP_36–44_ and PTHrP_102–111_ peptides efficiently induced peptide-specific cytotoxic T lymphocytes from peripheral blood mononuclear cells (PBMCs) of HLA-A24^+^ prostate cancer patients. Peptide-stimulated PBMCs showed cytotoxicity against prostate cancer cells in an HLA-A24-restricted manner. Experiments using antibodies and cold inhibition targets confirmed that their cytotoxicity was dependent on PTHrP peptide-specific and CD8^+^ T cells. Immunoglobulin G reactive to the PTHrP_102–111_ or PTHrP_110–119_ peptide was frequently detected in the plasma of prostate cancer patients, suggesting that the PTHrP_102–111_ peptide is able to elicit cellular and humoral immune responses in cancer patients. These results indicate that the PTHrP could be a promising target molecule for specific immunotherapy of HLA-A24^+^ prostate cancer patients with metastases.

Prostate cancer is one of the most common cancers among elderly men (Greenlee *et al*, 2000). Prostate cancer frequently metastasises to bone. Androgen withdrawal therapy has been applied for patients with bone metastases. Although hormone therapy can temporarily inhibit the progress of the disease in these patients, a progression to hormone-refractory prostate cancer inevitably occurs in most cases. Therefore, the development of new therapeutic modalities is needed.

Recent advances in tumour immunology have allowed us to identify the genes encoding human cancer-related antigens, and the epitopes, which are recognized by cytotoxic T lymphocytes (CTLs), in patients with various types of cancers (Boon *et al*, 1997; Rosenberg, 1999; Renkvist *et al*, 2001). The identified tumour antigens and their peptides have been applied for specific immunotherapy (Nestle *et al*, 1998; Rosenberg *et al*, 1998; Marchand *et al*, 1999). In the case of prostate cancer, tissue-specific antigens, which are expressed in the normal prostate, can also be target molecules for specific immunotherapy for patients with this disease. Immunotherapy targeting prostate-specific antigens or prostate-specific membrane antigens has been carried out, and antitumour effects have been observed in limited cases (Murphy *et al*, 1996; 1999; Tjoa *et al*, 1998; Small *et al*, 2000; Gulley *et al*, 2002).

Parathyroid hormone-related protein (PTHrP) is an autocrine or paracrine factor that binds to receptors on osteoblasts, and stimulates bone formation and reabsorption. Parathyroid hormone-related protein has limited homology with PTH at its NH2 terminus, and can bind to the same receptor as PTH, resulting in similar biological activity (Suva *et al*, 1987; Juppner *et al*, 1991). Parathyroid hormone-related protein plays a variety of physiological roles, including calcium transport, keratinocyte differentiation, smooth muscle relaxation, and cartilage development (Philbrick *et al*, 1996). In parathyroid cells, a high extracellular calcium concentration inhibits parathyroid hormone (PTH) secretion and the proliferation of parathyroid cells as a result of negative feedback regulation, whereas it evokes further PTHrP secretion and promotes worsening bone resorption (Sanders *et al*, 2001). Therefore, PTHrP has been considered to be responsible for the hypercalcemia associated with malignancy (Guise, 1997). In addition, prostate cancers have been reported to produce PTHrP (Francini *et al*, 2002). These lines of evidence suggest that PTHrP could be a promising target molecule for the immunotherapy of prostate cancer patients with bone metastases. In this study, we attempted to identify new, PTHrP-derived peptides that are immunogenic in HLA-A24^+^ prostate cancer patients.

## MATERIALS AND METHODS

### Patients

Informed consent was obtained from all of the HLA-A24^+^ prostate cancer patients and HLA-A24^+^ healthy volunteers who were enrolled in this study. None of the participants were infected with HIV. In total, 20 ml of peripheral blood was obtained, and the PBMCs were prepared by Ficoll-Conray density gradient centrifugation. The expression of HLA-A24 molecules on the PBMCs of the cancer patients and healthy donors was determined by flow cytometry.

### Cell lines

C1R-A24 is an HLA-A^*^2402-expressing subline of C1R lymphoma (Dr M Takiguchi, Kumamoto University, Japan). LNCaP is an HLA-A24 negative prostate cancer cell line. To establish LNCaP cells that stably express HLA-A24 molecules (designated as LNCaP-A24), an HLA-A^*^2402 gene was inserted into a pcDNA3.1/Hygro vector (Invitrogen, CA, USA), and electroporated into the LNCaP cell line (ATCC, Manassas, VA, USA), and selection was carried out with hygromycin B (Invitrogen) at a dose of 170 *μ*g ml^−1^. All cell lines were maintained in RPMI-1640 medium (Gibco BRL, Grand Island, NY, USA) supplemented with 10% FCS.

### Peptides

Four PTHrP-derived peptides (listed in Table 1

**Table 1:** Reactivity of PTHrP peptide-stimulated PBMCs from HLA-A24^+^ healthy donors and prostate cancer patients

	**Peptides**						
	**Name**	**PTHrP_36–44_**	**PTHrP_102–111_**	**PTHrP_25–34_**	**PTHrP_110–119_**	**Flu**	**EBV**
**PBMCs**	**Amino-acid sequence**	**RAVSEHQLL**	**RYLTQETNKV**	**RSVEGLSRRL**	**KVETYKEQPL**	**RFYIQMCTEL**	**TYGPVFMCL**
**Derived from**	**Score^a^**	**14.4**	**19.8**	**17.3**	**14.4**		
IFN-γ production (pg/ml)^b^							
*Healthy donors*							
#1		154	352	10	394	306	0
#2		156	132	8	17	0	207
#3		497	0	7	20	17	0
#4		0	0	37	2	59	14
#5		184	0	166	38	0	27
#6		1354	0	0	357	124	168
#7		166	38	0	0	1017	0
#8		0	194	0	1017	0	0
#9		0	5624	5	61	123	228
#10		0	168	1354	0	0	3
							
Total		6/10	5/10	2/10	3/10	4/10	3/10
							
*Cancer patients*							
#1		180	154	145	0	0	15
#2		122	138	15	9	5	0
#3		699	8	17	38	0	21
#4		31	105	24	19	0	159
#5		799	28	16	10	130	20
#6		500	4	1	14	198	15
#7		317	0	0	0	ND	ND
#8		4	1060	411	23	115	189
#9		17	101	1	0	709	3
#10		180	198	196	118	40	27
							
Total		7/10	6/10	3/10	1/10	4/9	2/9

a The score represents the estimated half-time of dissociation of the PTHrP peptides binding to HLA-A24 molecules.

b The PBMCs of HLA-A24^+^ healthy donors and prostate cancer patients were stimulated *in vitro* with the indicated PTHrP peptide, as described in Material and Methods. On the 15th day, the cultured PBMCs were tested for their reactivity to C1R-A24 cells, which were prepulsed with the corresponding peptide or the HIV peptide. The values represent the mean of two wells, and the background IFN-γ production in response to the HIV peptide was subtracted. Significant values (*P*<0.05 by two-tailed Student’s *t*-test) are underlined. ND=not done.

) were prepared based on the HLA-A24 binding motif (Parker *et al*, 1994; Rammensee *et al*, 1995). All peptides were of >90% purity and were purchased from Biologica Co., Nagoya, Japan. Influenza (Flu) virus-derived (RFYIQMCYEL), EBV-derived (TYGPVFMCL), and HIV-derived peptides (RYLRQQLLGI) with the HLA-A24 binding motif were used as controls. All peptides were dissolved with DMSO at a dose of 10 mg ml^−1^.

### Assay for peptide-specific CTLs in PBMCs

The assay for the detection of peptide-specific CTLs in PBMCs was performed according to a previously reported method (Hida *et al*, 2002). In brief, PBMCs (1 × 10^5^ cells per well) were incubated with 10 *μ*g ml^−1^ of each peptide in a U-bottom-type 96-well microculture plate (Nunc, Roskilde, Denmark) at a volume of 200 *μ*l of culture medium. The culture medium consisted of 45% RPMI-1640, 45% AIM-V medium (Gibco BRL), 10% FCS, 100 U ml^−1^ of IL-2, and 0.1 mM MEM nonessential amino-acid solution (Gibco, BRL). Half of the culture medium was removed and replaced with new medium containing a corresponding peptide (20 *μ*g ml^−1^) every 3 days. On the 15th day of culture, the cultured cells were separated into four wells; two wells were used for the PTHrP peptide-pulsed C1R-A24 cells, and the other two wells were used for the HIV peptide-pulsed C1R-A24 cells. After an 18-h incubation period, the supernatants were collected, and the level of IFN-*γ* was determined by ELISA (limit of sensitivity: 10 pg ml^−1^).

### Cytotoxicity assay

After *in vitro* stimulation with the PTHrP peptides, the peptide-stimulated PBMCs were additionally cultured with 100 U ml^−1^ IL-2 for approximately 10 days, in order to obtain a sufficient number of cells to carry out a cytotoxicity assay. These cells were then tested for cytotoxicity against both LNCaP and LNCaP-A24 by a 6-h ^51^Cr-release assay. A total of 2000 ^51^Cr-labelled cells per well were cultured with effector cells in 96-round-well plates at the indicated effector/target ratios. In some experiments, either anti-HLA class I (W6/32: mouse IgG2a), anti-HLA-DR (L243: mouse IgG2a), anti-CD4 (NU-TH/I: mouse IgG1), anti-CD8 (NU-TS/C: mouse IgG2a), or anti-CD14 (H14: mouse IgG2a) mAb was added to the wells at a dose of 20 *μ*g ml^−1^ at the initiation of the assay.

### Cold inhibition assay

The specificity of the PTHrP peptide-stimulated CTLs was confirmed by a cold inhibition assay. In brief, ^51^Cr-labelled target cells (2 × 10^3^ cells per well) were cultured with the CTLs (4 × 10^4^ cells per well) in 96-round-well plates with 2 × 10^4^ cold target cells. C1R-A24 cells, which were prepulsed with either the HIV peptide or a corresponding PTHrP peptide, were used as cold targets.

### Detection of peptide-specific IgG

The peptide-specific IgG levels in the plasma were measured by ELISA, as previously reported (Nakatsura *et al*, 2002; Ohkouchi *et al*, 2002). In brief, peptide (20 *μ*g per well)-immobilised plates were blocked with Block Ace (Yukijirushi, Tokyo, Japan) and washed with 0.05% Tween-20-PBS, after which 100 *μ*l per well of plasma sample diluted with 0.05% Tween-20-Block Ace was added to the plate. After a 2-h incubation at 37°C, the plates were washed and further incubated for 2-h with a 1 : 1000-diluted rabbit anti-human IgG (*γ*-chain-specific) (DAKO, Glostrup, Denmark). The plates were washed, and then 100 *μ*l of 1 : 100-diluted goat anti-rabbit IgG-conjugated horseradish peroxidase (EnVision, DAKO) was added to each well, and the plates were then incubated at room temperature for 40 min. After the plates were washed once, 100 *μ*l per well of tetramethyl benzidine substrate solution (KPL, Guildford, UK) was added, and the reaction was stopped by the addition of 1 M phosphoric acid. The values are shown as optical density (OD) units ml^−1^. IgG reactive to a corresponding PTHrP peptide was judged to be positive when the difference of the OD in 1 : 100-diluted plasma exceeded 0.05. To confirm the specificity of IgG to the indicated PTHrP peptide, sample plasma was cultured with plates coated with either the corresponding PTHrP peptide or an irrelevant PTHrP peptide. Thereafter, the levels of PTHrP peptide-specific IgG in the resulting supernatant were determined by ELISA.

### Statistics

The statistical significance of the data was determined using a two-tailed Student’s *t*-test. A *P*-value of less than 0.05 was considered to be statistically significant.

## RESULTS

### Induction of PTHrP peptide-specific CTLs from HLA-A24^+^ healthy donors and prostate cancer patients

**Figure 1:**
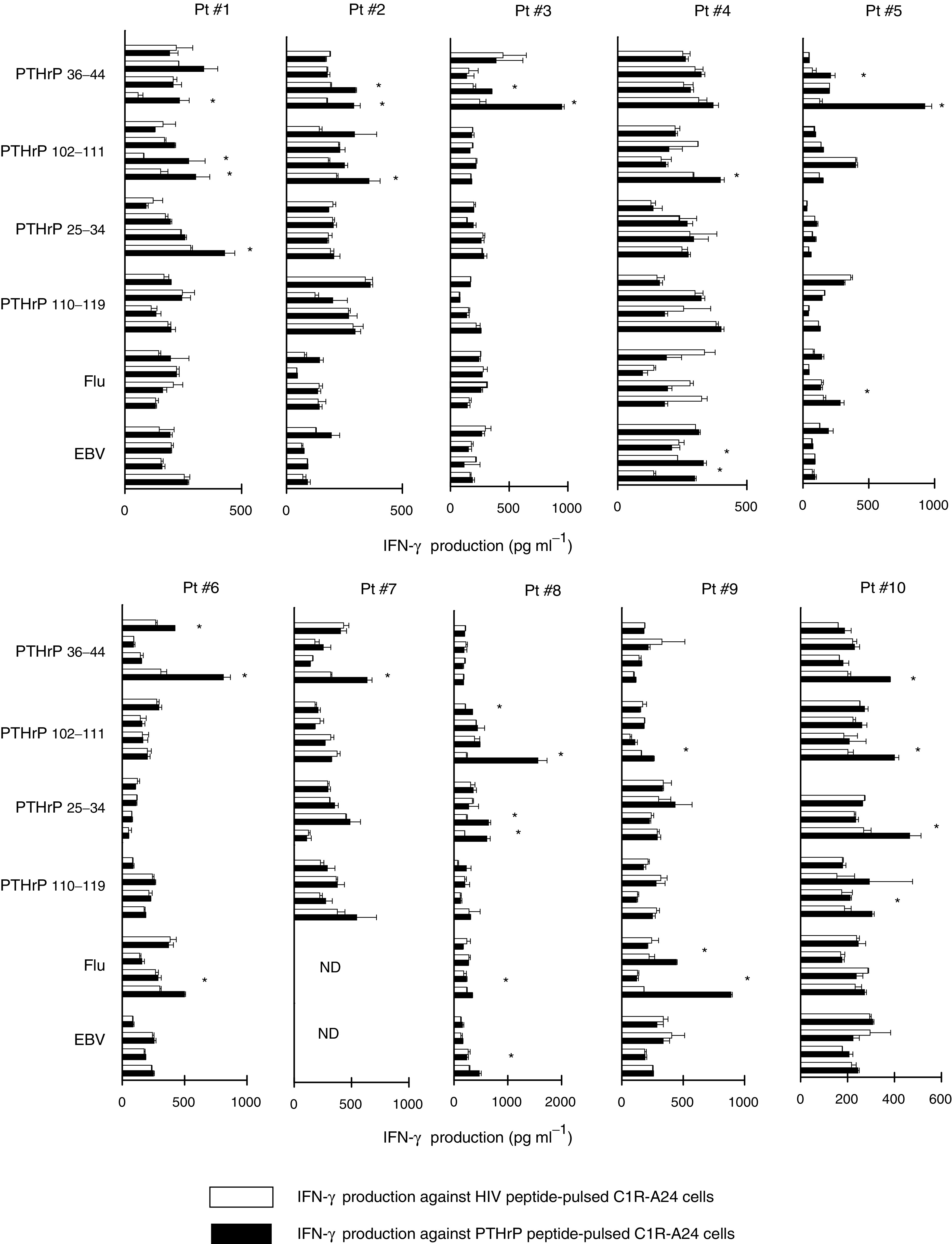
Induction of PTHrP peptide-specific CTLs from the PBMCs of HLA-A24^+^ prostate cancer patients. PBMCs from 10 HLA-A24^+^ prostate cancer patients were stimulated *in vitro* with the PTHrP peptides indicated, as described in Materials and Methods. On the 15th day, the peptide-stimulated cells were cultured with C1R-A24 cells, which were prepulsed with an HIV peptide (open bar) and the indicated PTHrP peptide (closed bar) for 18-h. The levels of IFN-*γ* in the supernatants were then determined by ELISA. ^*^P<0.05 was considered statistically significant.

First, four PTHrP-derived peptides were prepared based on their binding affinity to HLA-A24 molecules (Parker *et al*, 1994; Rammensee *et al*, 1995) (Table 1). Although the PTHrP_1–36_ peptide is a propeptide (Suva *et al*, 1987; Juppner *et al*, 1991), the PTHrP_25–34_ peptide was included. With regard to the difference in amino acids, three amino acids were found to differ between the PTHrP_36–44_ peptide and PTH, and all of the amino acids were found to differ between the other three PTHrP peptides and PTH. Next, to investigate the immunogenicity of these four PTHrP peptides, the PBMCs of 10 HLA-A24^+^ healthy donors and 10 HLA-A24^+^ prostate cancer patients were stimulated with each of four PTHrP peptides, and were then examined for their IFN-*γ* production in response to C1R-A24 cells, which were prepulsed with either a corresponding PTHrP peptide or the HIV peptide (Table 1). Flu- and BEV-derived peptides were used as controls. The assay was carried out in quadruplicate. The cultured cells in one well were separated into four wells. Two wells were used for the PTHrP peptide-pulsed C1R-A24 cells, and the other two wells for the HIV peptide-pulsed C1R-A24 cells. The background IFN-*γ* production in response to the HIV peptide was subtracted, and the results that showed the best response are shown in Table 1. The successful induction of peptide-specific CTLs was judged to be positive when significant values (*P*<0.05 by two tailed Student’s *t*-test) were observed. The results showed that the PTHrP_36–44_, PTHrP_102–111_, PTHrP_25–34_, and PTHrP_110–119_ peptides induced peptide-specific CTLs in six, five, two, and three of 10 HLA-A24^+^ healthy donors, respectively. These PTHrP peptides also induced peptide-specific CTLs in seven, six, three, and one of 10 HLA-A24^+^ prostate cancer patients, respectively. The net IFN-*γ* production of the cases with 10 HLA-A24^+^ prostate cancer patients in response to the corresponding PTHrP peptide or the HIV peptide are shown in Figure 1

. In total, these findings indicate that both the PTHrP_36–44_ and PTHrP_102–111_ peptides are promising candidates to generate peptide-specific CTLs from HLA-A24^+^ prostate cancer patients.

### Induction of prostate cancer-reactive CTLs using PTHrP_36–44_ and PTHrP_102–111_ peptides

**Figure 2:**
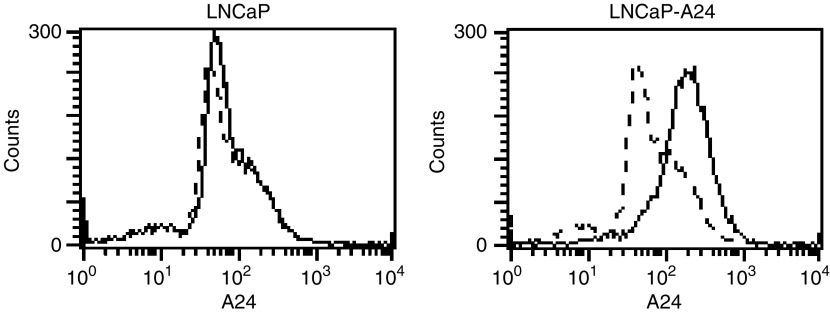
An HLA-A24-expressing LNCaP cell line. Flow cytometric analysis was performed on the LNCaP and LNCaP-A24 cells. These cells were stained with anti-HLA-A24 mAb, followed by FITC-conjugated anti-mouse IgG mAb. The dotted lines represent staining without the first mAb.

**Figure 3:**
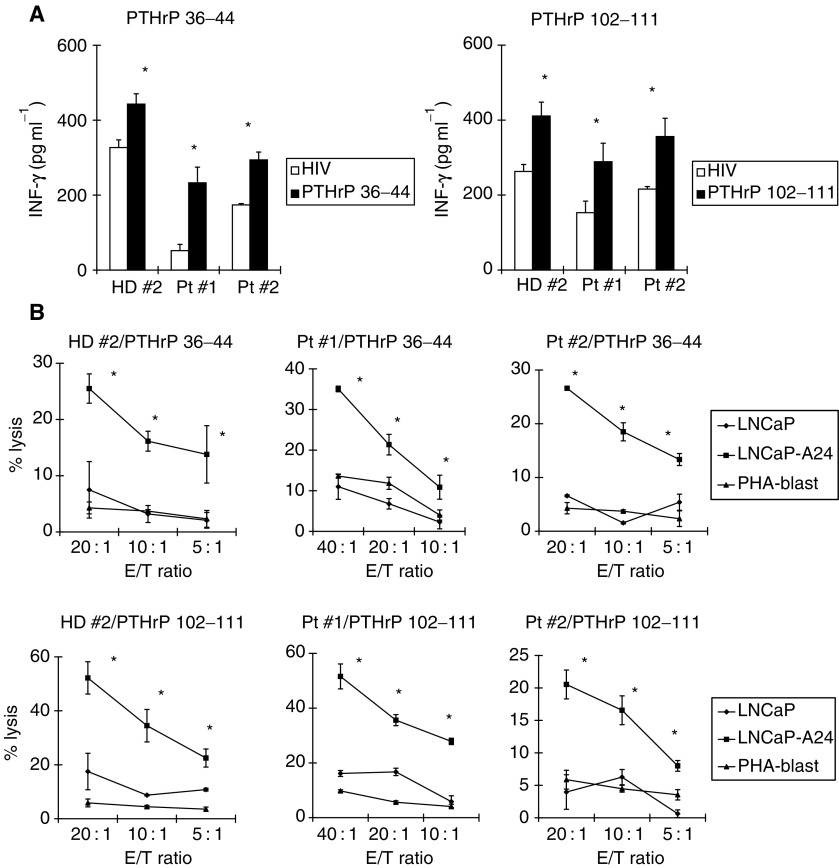
Induction of HLA-A24-restricted and prostate cancer-reactive CTLs from the PBMCs of healthy donors and cancer patients. (**A**) PBMCs from one HLA-A24^+^ healthy donor (HD #2) and from two HLA-A24^+^ prostate cancer patients (Pt #1 and Pt #2) were stimulated *in vitro* with the indicated PTHrP peptides, as described in Materials and Methods. On the 15th day, half of the cultured cells were harvested, pooled from four wells, and cultured with C1R-A24 cells, which were prepulsed with an HIV peptide (open symbol) and the indicated PTHrP peptide (closed symbol) for 18-h. The levels of IFN-*γ* in the supernatants were then determined by ELISA. (**B**) Thereafter, these cells were examined for their cytotoxicity against the LNCaP cells (HLA-A24^-^), LNCaP-A24 cells (HLA-A24^+^), and PHA-blastoid T cells (HLA-A24^+^). A 6-h ^51^Cr-release assay was performed. Values represent the mean of triplicate assays. ^*^P<0.05 was considered statistically significant.

**Figure 4:**
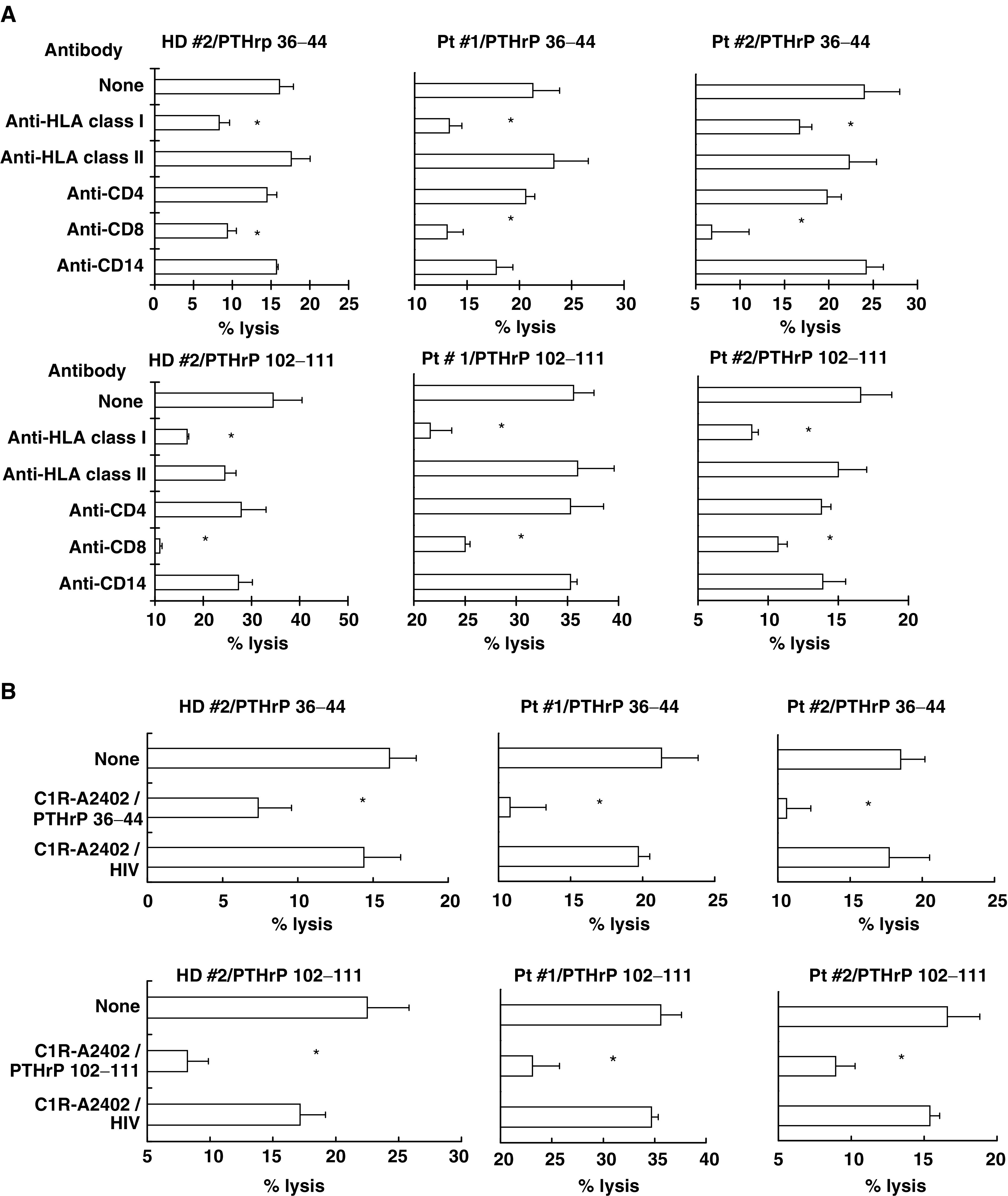
CD8^+^ T-cell-dependent and PTHrP peptide-specific cytotoxicity against LNCaP-A24 cells. (**A**) The PTHrP peptide-stimulated PBMCs, described in Figure 2, were examined for their cytotoxicity against the LNCaP-A24 cell line, with or without anti-HLA class I, anti-HLA class II, anti-CD4, anti-CD8, or anti-CD14 mAb at a dose of 20 *μ*g ml^−1^. The values represent the mean of triplicate assays. ^*^P<0.05 was considered statistically significant. (**B**) The cytotoxicity against the LNCaP-A24 cell line (2 × 10^3^ cells per well) was also examined in the presence of unlabelled C1R-A24 cells (2 × 10^4^ cells per well), which were prepulsed with the HIV peptide or a corresponding PTHrP peptide. The values represent the mean of triplicate assays. ^*^P<0.05 was considered statistically significant.

In order to investigate the HLA-A24-restricted and prostate cancer-reactive cytotoxicity of peptide-stimulated PBMCs, we prepared an HLA-A24-expressing LNCaP cell line, which we designated LNCaP-A24 (Figure 2

). LNCaP has previously been reported to produce PTHrP (Francini *et al*, 2002). A parental LNCaP cell line was negative for the cell surface expression of HLA-A24 molecules, whereas the LNCaP-A24 cell line expressed HLA-A24 molecules on their cell surface. It was then determined whether PBMCs stimulated by either the PTHrP_36–44_ or PTHrP_102–111_ peptide could induce prostate cancer-reactive CTLs from HLA-A24^+^ healthy donors and prostate cancer patients. PBMCs from HLA-A24^+^ healthy donors and cancer patients were repeatedly stimulated with the indicated PTHrP peptide, based on the culture protocol described in Materials and Methods. After confirming that these peptide-stimulated cells could produce IFN-*γ* in response to PTHrP peptide-pulsed C1R-A24 cells, the peptide-stimulated PBMCs were examined for their cytotoxicity against three targets. It was found that the PTHrP peptide-stimulated PBMCs from HD#2, Pt#1, and Pt#2 produced higher levels of IFN-*γ* in response to the corresponding PTHrP peptide-pulsed C1R-A24 cells than to the HIV peptide-pulsed C1R-A24 cells (Figure 3A

). These peptide-stimulated PBMCs also showed higher levels of cytotoxicity against the LNCaP-A24 cell line than against the LNCaP line and HLA-A24^+^ PHA-induced T cell blasts (Figure 3B). In addition, their cytotoxicity against LNCaP-A24 was significantly inhibited by the addition of anti-HLA-class I and anti-CD8 mAbs, but not by the addition of other anti-HLA-class II, anti-CD4, or anti-CD14 mAbs (Figure 4A

). Furthermore, their cytotoxicity against the LNCaP-A24 cell line was significantly suppressed by the addition of the corresponding PTHrP peptide-pulsed C1R-A24 cells, as a cold target, but this suppression was not observed with the addition of HIV peptide-pulsed C1R-A24 cells (Figure 4B). In addition, we observed that these PTHrP peptide-stimulated PBMCs from cancer patients showed cytotoxicity against another prostate cancer cells PC-93-A24, stably expressed the HLA-A24 molecules and produced PTHrP (data not shown). These results indicate that both the PTHrP_36–44_ and PTHrP_102–111_ peptides have the potential to induce prostate cancer-reactive CTLs from HLA-A24^+^ prostate cancer patients, and that their cytotoxicity against prostate cancer was dependent on PTHrP peptide-specific CD8^+^ T cells.

### Detection of IgG reactive to the PTHrP peptides

We previously reported that IgGs reactive to CTL epitope peptides were detected in healthy donors and cancer patients (Nakatsura *et al*, 2002; Ohkouchi *et al*, 2002). IgGs reactive to prostate-related antigens were also detected in healthy donors and prostate cancer patients (Harada *et al*, 2003a; Kobayashi *et al*, 2003; Matsueda *et al*, 2004) Therefore, we attempted to determine whether IgG reactive to four PTHrP-derived peptides could be detected in the plasma of cancer patients and healthy donors. The result was that IgG reactive to either the PTHrP_102–111_ or the PTHrP_109–119_ peptide was detected in eight of 10 healthy donors and in seven of 10 prostate cancers (Table 2

**Table 2:** IgG reactive to the PTHrP peptides in plasma of HLA-A24^+^ healthy donors and prostate cancer patients

	**Healthy donors**											**Prostate cancer patients**										
**Peptides**	**#1**	**#2**	**#3**	**#4**	**#5**	**#6**	**#7**	**#8**	**#9**	**#10**	**Total**	**#1**	**#2**	**#3**	**#4**	**#5**	**#6**	**#7**	**#8**	**#9**	**#10**	**Total**
PTHrP_36–44_	−	−	−	−	−	−	+	+	+	−	3/10	−	−	−	−	+	−	−	−	−	−	1/10
PTHrP_102–111_	+	−	+	+	+	−	+	+	+	+	8/10	+	+	−	−	+	+	+	+	+	−	7/10
PTHrP_25–34_	−	−	−	−	−	−	−	−	−	−	0/10	−	−	−	−	−	−	−	−	−	−	0/10
PTHrP_110–119_	+	+	+	−	+	−	+	+	+	+	8/10	+	+	−	−	−	+	+	+	+	+	7/10

**Figure 5:**
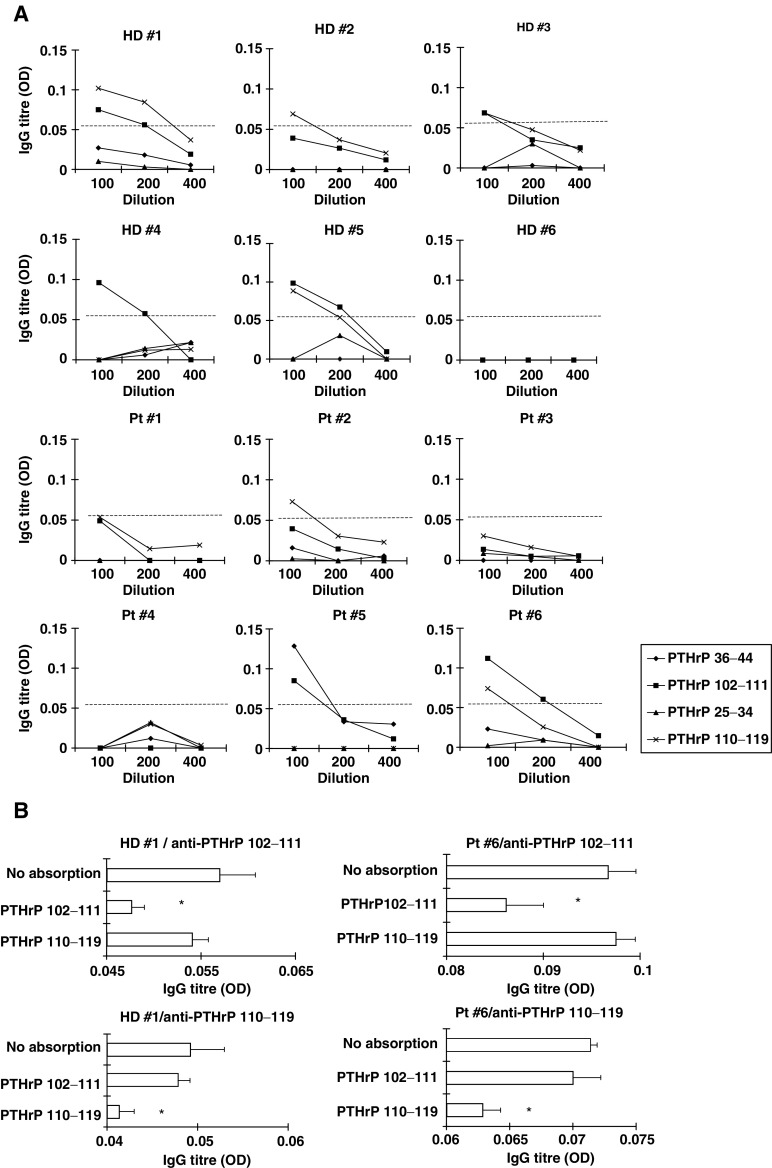
IgG reactive to the PTHrP peptides in plasma from healthy donors and prostate cancer patients. (**A**) Representative results from six healthy donors and six prostate cancer patients are shown. These values are shown as optical density (OD), and the responses to the HIV peptide were subtracted. IgG reactive to a corresponding PTHrP peptide was judged to be positive when the difference of the OD in 1 : 100-diluted plasma exceeded 0.05. The cutoff level (OD: 0.05) was determined based on the levels of anti-HIV peptide IgG in HIV-negative healthy donors. (**B**) To confirm the specificity of IgG to the indicated PTHrP peptides, 100 *μ*l of sample plasma from either HD #1 and Pt #6 was cultured in a plate precoated with either a corresponding PTHrP peptide or an irrelevant PTHrP peptide. Thereafter, the levels of IgG reactive to the PTHrP_102–111_ peptide or the PTHrP_110–119_ peptide in the resultant samples were determined by ELISA.

IgG reactive to the corresponding peptide was judged to be positive when the difference in the OD in 1 : 100-diluted plasma exceeded 0.05. The cutoff level (OD: 0.05) was determined based on the levels of anti-HIV peptide IgG in HIV-negative healthy donors.

). Representative results are in Figure 5A

. However, IgG reactive to the PTHrP_36–44_ peptide was detected in three of 10 healthy donors and one of 10 prostate cancer patients, respectively. No IgG reactive to PTHrP_25–34_ was detected in any of the healthy donors or cancer patients. The levels of PTHrP peptide-specific IgG were significantly diminished by culturing the plasma in the corresponding PTHrP peptide-coated wells (Figure 5B). This peptide-specific absorption demonstrated the validity of the present assay system.

## DISCUSSION

Prostate cancer appears to be a good target for the development of specific immunotherapies (Harada *et al*, 2003b). In recent years, our group has attempted to identify epitope peptides derived from prostate-related antigens that would be able to generate prostate cancer-reactive CTLs from prostate cancer patients (Inoue *et al*, 2001; Kobayashi *et al*, 2003; Harada *et al*, 2003a; Matsueda *et al*, 2004). However, one major obstacle encountered when treating prostate cancer patients is the treatment of bone metastases, as prostate cancer frequently metastasises to the bone tissue. Therefore, we undertook the present study to identify epitope peptides that could potentially be suitable for specific the immunotherapy of HLA-A24^+^ prostate cancer patients with metastases.

PTHrP is known to be a key agent in the development of bone metastasis in cases of prostate cancer, and prostate cancer cells has been reported to produce PTHrP (Francini *et al*, 2002). These lines of evidence indicate that PTHrP could be a good target for the development of specific immunotherapies against metastatic prostate cancer. Indeed, PTHrP_59–68_ and PTHrP_165–173_ peptides have been reported to be candidates for such specific immunotherapy of HLA-A2^+^ prostate cancer patients (Guise, 1997; Francini *et al*, 2002). In this study, we identified new PTHrP peptides that have the potential to generate prostate cancer-specific CTLs in HLA-A24^+^ prostate cancer patients, in order to extend the possibility of PTHrP peptide-based anticancer vaccine. We revealed that both the PTHrP_36–44_ and the PTHrP_102–111_ peptides have the potential to induce prostate cancer-reactive CTLs in HLA-A24^+^ prostate cancer patients. PBMCs from HLA-A24^+^ prostate cancer patients showed peptide-specific IFN-*γ* production in six or seven of 10 patients when stimulated with the PTHrP_102–110_ and PTHrP_36–44_ peptide, respectively. More importantly, PBMCs that were stimulated with these PTHrP peptides showed cytotoxicity against prostate cancer cells in an HLA-A24-restricted manner. These results indicate that these two PTHrP peptides are immunogenic, and therefore potentially useful for the specific immunotherapy of HLA-A24^+^ prostate cancer patients with metastases.

The PTHrP_36–44_ and the PTHrP_102–110_ peptides also induced peptide-specific and tumour-reactive CTLs from the PBMCs of HLA-A24^+^ healthy donors. This result is consistent with that of a previous report demonstrating the induction of PTHrP peptide-specific CTLs from the PBMCs of HLA-A2^+^ healthy donors (Francini *et al*, 2002). As the PTHrP_36–44_ peptide shares three amino acids with PTH, and because there is no homology between the PTHrP_102–111_ peptide and PTH, crossreactivity between the PTHrP peptides and PTH could be excluded. Low levels of PTHrP have been sporadically detected in keratinocytes, uterus, and mammary glands during lactation (Tian *et al*, 1993). Recent advances in tumour immunology have revealed that self-antigens on human cancer cells are the most prevalent antigens recognized by the immune system (Rosenberg, 1999; Renkvist *et al*, 2001). CTL precursors reactive to nonmutated self-antigens may circulate in the peripheral blood of both certain healthy donors and cancer patients.

Here, we investigated whether or not IgG against PTHrP peptides would be detectable in plasma from HLA-A24^+^ healthy donors and prostate cancer patients, because the antibodies against CTL epitope peptides had already been observed in certain cancer patients and healthy donors (Nakatsura *et al*, 2002; Ohkouchi *et al*, 2002). We also previously reported that IgG reactive to peptides derived from prostate-related antigens was frequently detectable in healthy donors and prostate cancer patients (Harada *et al*, 2003a; Kobayashi *et al*, 2003; Matsueda *et al*, 2004). In this study, IgG reactive to either the PTHrP_102–111_ peptide or PTHrP_110–119_ peptide was frequently detected in healthy donors as well as in prostate cancer patients. This means that the PTHrP_102–111_ peptide was recognized by both the cellular and humoral immune systems. Although we do not yet have a clear understanding of the roles played by peptide-specific IgG in antitumour immune responses, our clinical trials revealed that a peptide vaccination frequently resulted in the induction of IgG reactive to the CTL epitope peptides which were administered (Noguchi *et al*, 2003; Tanaka *et al*, 2003). In addition, the induction of IgG reactive to the vaccinated peptides was positively correlated with longer survival of advanced lung cancer patients (Mine *et al*, 2003). As regards the use of a peptide vaccination in cases of gastric cancer, prolonged survival has been observed in patients showing not only cellular, but also humoral immune responses to vaccinated peptides (Sato *et al*, 2003). In addition, the induction of IgG reactive to the administered peptides was correlated with a clinical response among patients with recurrent gynecologic cancer (Tsuda *et al*, 2004). Furthermore, we recently analysed 113 vaccinated patients with various types of cancers, and revealed that the augmentation of peptide-specific IgG after peptide vaccination could be a laboratory marker for the prediction of prolonged survival in vaccinated cancer patients compared to the induction of peptide-specific CTLs or the delayed-type hypersensitivity test (Mine *et al*, 2004). Moreover, we recently observed that peptide vaccination with a 9-mer CTL epitope peptide could induce peptide-specific and HLA-DR-restricted CD4^+^ T cells *in vivo* (Harada *et al*, 2004). As these findings provide circumstantial evidence, further clinical study is needed to elucidate the role and meaning of peptide-specific IgG in anticancer immunotherapy.

In conclusion, we identified new two PTHrP-derived peptides that are immunogenic in HLA-A24^+^ prostate cancer patients. The frequencies of the HLA-A24 allele are relatively high throughout the world (Imanishi *et al*, 1992). The information provided here might increase the possibility of treating HLA-A24^+^ prostate cancer patients with metastases using peptide-based immunotherapy.
